# Flaxseed modulates inflammatory and oxidative stress biomarkers in cystic fibrosis: a pilot study

**DOI:** 10.1186/s12906-015-0651-2

**Published:** 2015-05-13

**Authors:** Jason B Turowski, Ralph A Pietrofesa, John A Lawson, Melpo Christofidou-Solomidou, Denis Hadjiliadis

**Affiliations:** Division of Pulmonary, Allergy, and Critical Care Medicine, Department of Medicine, University of Pennsylvania, Hospital of the University of Pennsylvania, 835W Gates Building, 3600, Spruce Street, Philadelphia, PA 19104 USA; Department of Pharmacology, University of Pennsylvania Perelman School of Medicine, Philadelphia, PA 19104 USA

**Keywords:** Antioxidant, Biomarker, Cystic fibrosis, Cytokine, Enterodiol, Enterolactone, Flaxseed, Inflammation, Lignan, Secoisolariciresinol diglucoside

## Abstract

**Background:**

Cystic fibrosis (CF) leads to advanced lung disease despite aggressive care. Persistent inflammation and oxidative stress contribute to exacerbations and disease progression. Flaxseed (FS), a dietary botanical supplement with high fiber, lignan phenolics, and omega-3 fatty acids has anti-inflammatory and antioxidant properties in murine models of acute and chronic lung injury. This pilot study was designed to determine whether CF patients could tolerate FS, evaluate circulating FS metabolites, and study biomarkers of lung damage, as a prelude to studying clinical outcomes.

**Methods:**

10 CF patients and 5 healthy volunteers consumed 40 g of FS daily for 4 weeks with safety and tolerability being assessed. Urine was evaluated for systemic oxidative stress and plasma for FS metabolites (enterolignans) and cytokine levels. Buccal swabs were analyzed for gene expression of Nrf2-regulated antioxidant enzymes including Heme Oxygenase-1 (HO-1) and NAD(P)H Quinone Oxidoreductase 1 (NQO1).

**Results:**

All subjects completed the study without serious adverse events. Plasma levels of enterolignans were detectable in both healthy controls and CF volunteers. CF patients were stratified based on plasma enterolignan levels after 2 weeks of FS administration into high- (174 to 535 nM ED and 232 to 1841 nM EL) and low- (0 to 32 nM ED and 0 to 40 nM EL) plasma lignan cohorts. The low enterolignan level cohort experienced a statistically significant drop in urinary inflammatory IsoP and plasma TNFα levels, while demonstrating higher average NQO1 mRNA levels in buccal epithelium compared to high-lignan patients.

**Conclusions:**

This pilot study demonstrated that FS is tolerated by CF patients. FS metabolites could be detected in the plasma. Future studies will assess appropriate dosing and target populations for FS, while exploring clinical outcomes.

**Trial registration:**

ClinicalTrials.gov identifier: NCT02014181.

## Background

Cystic fibrosis results from malfunction of the cystic fibrosis transmembrane conductance regulator (CFTR) responsible for chloride transport; this therefore affects the viscosity of mucus covering epithelia throughout the body [1-3]. Dysfunction contributes to chronic sinusitis, pancreatic insufficiency, and pulmonary infections with virulent pathogens including *Pseudomonas aeruginosa* and methicillin-resistant *Staphylococcus aureus* [4,5]. Exacerbations are characterized by inflammation measured in the blood, urine, and sputum [6-8].

Despite enzyme replacement, antibiotics, and broncho-pulmonary hygiene – standard practice helping to improve life expectancy in CF, exacerbations still occur and as lung function declines, quality of life worsens and early death is common. Dietary antioxidants have shown promise when used in cardiovascular diseases [9]; but their use has never been validated in CF.

Christofidou-Solomidou has investigated the use of antioxidants in acute and chronic lung injury pioneering animal models of acute and chronic systemic oxidative stress to evaluate the anti-inflammatory/antioxidant potential of naturally occurring phytochemicals [10-15]. Flaxseed (FS), a wholegrain rich in omega-3 fatty acids and lignans demonstrates potent anti-inflammatory, antioxidant, and anti-fibrotic properties in murine models of ischemia-reperfusion injury (IRI) and radiation-induced lung injury (RILI) [10,12,14]. Omega-3 fatty acids reduce inflammation [16], while lignans are potent antioxidant phytoestrogens [17]. FS has been well tolerated in studies involving patients with diabetes or prostate cancer [18,19].

Patients with CF are often excluded from dietary studies because investigators hypothesize malabsorption might negate beneficial properties of the study compound. Serum levels of FS metabolites – enterodiol (ED) and enterolactone (EL) can be sustained in healthy volunteers [20,21] and we aimed to demonstrate this with our CF cohort.

FS in murine models induces the expression of phase II detoxification and antioxidant enzymes (Heme Oxygenase 1 (HO-1) and NAD(P)H Quinone Oxidoreductase 1 (NQO1)), transcriptionally regulated by Nrf2 binding to the antioxidant response element (ARE) [15]. Validated biomarkers of systemic oxidative stress, 8,12-iso-iPF2α-VI isoprostane (IsoP) and DNA damage, 8-oxo-7,8-dihydro-2′deoxyguanosine (8-oxo-dGuo) have been utilized to quantify inflammation and oxidative stress in diseases including CF and were quantified here as secondary outcome measures [22-25].

We wanted to know if FS is safe and tolerable in stable CF adults as compared to healthy volunteers. We secondarily hypothesized that FS with its beneficial properties would increase antioxidant gene expression. Lastly, we hoped to measure biomarkers of chronic inflammation and oxidative stress in urine and plasma. This is the first study to investigate the effect of FS on inflammation and oxidative stress in patients with CF. Hopefully it will lead to future studies that can demonstrate improvement in clinically relevant outcomes, including enhanced nutrition and lung function, and decreased exacerbations.

## Methods

### Experimental design

All fifteen subjects were enrolled to consume 40 grams of finely ground wholegrain FS for one month provided in resealable individual pouches. On the first visit prior to starting FS, blood, urine and buccal swab samples were collected to establish baseline values of FS metabolite levels, biomarkers of oxidative stress and DNA damage, as well as baseline levels of gene expression of antioxidants HO-1 and NQO1.

Each patient consumed 40 grams of finely-ground flaxseed daily in the manner of their choice, typically as a primary ingredient in a smoothie. Patients returned each week to supply blood and urine samples to trend biomarkers of oxidative stress and trend levels of FS metabolites enterodiol and enterolactone, a metabolism requiring intact gut flora. If no side effects related to FS ingestion were recorded by the patient, one week during that month could be skipped in terms of supplying samples for the study – but FS was continued during that time. After one month on FS, the patient again returned to the clinic to supply blood, urine and now a follow up buccal swab to establish if after one month of FS, endogenous levels of antioxidant enzymes were boosted by FS administration. Lastly, after another month now off of FS, each patient was asked to return to the clinic to supply blood, urine, and buccal swabs to this time establish if off the supplement, biomarkers of oxidative stress and inflammation, as well as lignan levels, and endogenous antioxidant enzyme expression reverted back to baseline levels originally collected approximately 2 months prior.

### Enrollment criteria and outcome variables

**Figure 1:**
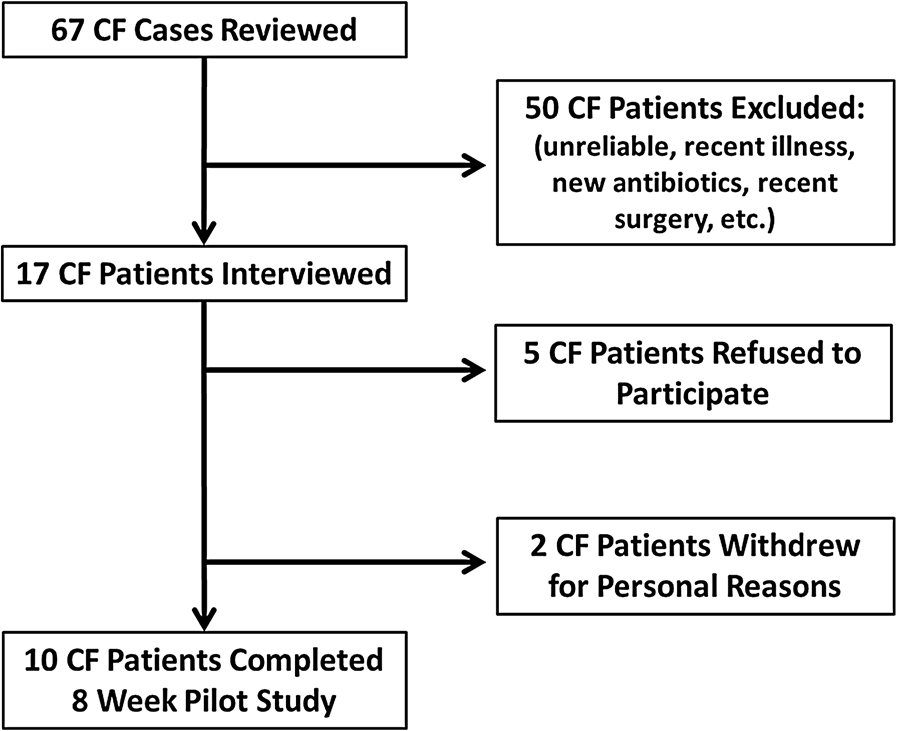
Study Enrollment Flow Diagram. Study enrollment opened July 2012. 67 CF cases were reviewed to determine eligibility. All cases were reviewed by 3 board-certified pulmonary and critical care physicians actively caring for adults with CF. 50 patients immediately met pre-determined exclusion criteria: actively infected, actively on intravenous antibiotics, recently had surgery, had gut issues or were known to be unreliable (>3 office no shows). 17 CF patients met eligibility criteria so were interviewed during the 3-month open enrollment period. Another 5 patients refused to participate due to distance from Philadelphia, concerns about meeting the requirements of the study, or were just not interested. Among the 12 patients that consented and began the trial, another 2 withdrew for personal reasons. Between July – November 2012, 10 CF patients completed the study.

This feasibility-pilot study was conducted at the University of Pennsylvania in accordance and after approval by the Institutional Review Board of the same institution, which approved the study prior to its initiation. Figure 1 displays patient enrollment and exclusions. 10 adult patients were recruited from the Adult CF Center and provided informed consent. Exclusion criteria included prior or planned hospitalization or surgical procedure within 1 month of enrollment (other than simple dental procedure); an acute pulmonary exacerbation; history of bowel resection, inflammatory bowel disease or distal intestinal obstruction syndrome; receiving broad spectrum intravenous or oral antibiotics (other than maintenance oral azithromycin or inhaled antibiotics) within 1 month of enrollment; current supplementation with FS or soy derivatives or allergies to them; active or prior ingestion of Vitamin E exceeding 30 IU within 21 days; significant liver disease (cirrhosis); significant renal dysfunction (GFR below 50 ml/hr/m^2^); or poorly controlled diabetes (evidenced by HgbA1c > 7.5%).

5 healthy adult volunteers from the University of Pennsylvania research and clinical community were recruited and provided informed consent to serve as controls. They were generally healthy without major medical problems, as determined by review of their past medical history by 2 co-investigators. No study volunteers could exceed age 65 or have extremes of BMI (<18 or >35).

**Figure 2:**
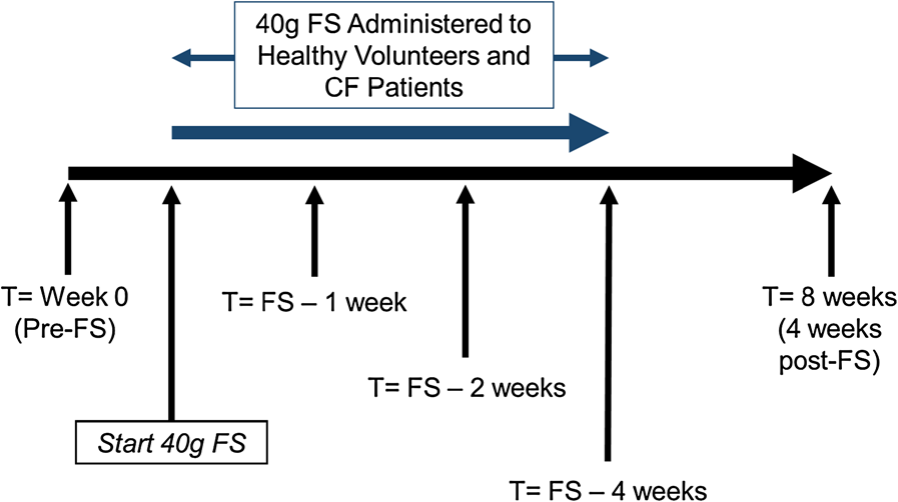
Study Schematic. Total duration of the study was 8 weeks. Patients (10 adult cystic fibrosis patients and 5 healthy volunteers) were enrolled at T = 0 at which time blood, urine and buccal swabs were collected to establish baseline levels of lignans, cytokines, isoprostanes, and 8-oxo-dGuo. Urine and blood samples were drawn during each visit at 1, 2, 4, and 8 weeks. Buccal swabs were performed at weeks 0, 4, and 8 (4 weeks post-FS).

Primary outcome was feasibility, toxicity, and tolerability of dietary FS administration in CF as documented through weekly surveys conducted at clinic - assessing side effects, serious adverse events, and favorable effects of FS. Secondary outcomes included measurements of urinary biomarkers of systemic oxidative stress and plasma levels of FS metabolites enterodiol (ED) and enterolactone (EL). Blood and urine samples were obtained at 5 time points (Figure 2): 1) Baseline – prior to FS; 2) FS – 1 Week; 3) FS – 2 Weeks; 4) FS – 4 Weeks and 5) 4 Weeks post-FS supplementation. Subjects consumed FS in a manner of their choice. Urine was analyzed for F2-IsoP and 8-oxo-dGuo levels. Plasma was analyzed for FS metabolites and systemic cytokines. A buccal swab was obtained at baseline, after 4 weeks of FS supplementation, and at 4 weeks post-FS to determine antioxidant gene expression.

### Managing human tissue samples

The plasma specimens were aliquoted and stored at −80°C until lignan measurements could be performed. 5 ml of blood was obtained prior to starting FS, approximately 15–20 ml of blood was obtained while the patient ingested FS, and 5 ml of blood was obtained after a one-month period of washout when no FS had been ingested, for a total of approximately 30 ml of blood obtained during the pilot. Urine samples were collected, centrifuged at 1500 RPM (297*g force) for 5 min to settle debris and also stored at −80°C until measurements of oxidation and inflammation could be performed.

Buccal samples were collected using a MasterAmp Buccal Swab Brush by Epicentre Technologies of Madison, WI. The samples gathered by each brush tip were deposited into an RNA*later* 1.5 ml container by Ambion. The combined buccal swab specimen in the room temperature RNA*later* vessel was transported on ice, initially stored at 4°C in the laboratory refrigerator for about one week to acclimatize the sample. The sample was then stored at −80°C until RT-PCR was performed, a storage period of under one month typically.

### Quantifying FS lignan metabolites and biomarkers of oxidative stress

Circulating plasma levels of ED and EL at the five specimen time points were determined by liquid chromatography tandem mass spectrometry (LC/MS/MS) as previously described [14,26] using commercially available standards in 95% purity (Chromadex, Inc., Santa Ana, CA). Urinary 8,12-iso-iPF2α-VI isoprostane levels [22] and 8-oxo-7,8-dihydro-2′-deoxy-guanosine (8-oxo-dGuo) levels [27] were measured and normalized to urine creatinine level. 8-oxo-dGuo levels were measured by high-performance liquid chromatography-electrospray tandem mass spectrometry [23].

### Gene expression analysis

Quantitative Reverse Transcription Polymerase Chain Reaction (qPCR) was performed using TaqMan® Probe-Based Gene Expression Assays supplied by Applied Biosystems, Life Technologies (Carlsbad, CA). Individual Taqman gene expression assay was selected for HO-1 and NQO1. Cells were lysed and RNA was isolated using a commercially available kit, QIAprep Spin Miniprep Kit, supplied by Qiagen (Valencia, CA). Total RNA was quantified using a NanoDrop 2000 (ThermoFisher Scientific, Waltham, MA). Reverse transcription of RNA to cDNA was then performed on a Veriti® Thermal Cycler using the High Capacity RNA to cDNA kit supplied by Applied Biosystems, Life Technologies (Carlsbad, CA). qRT-PCR was performed using 25 ng of cDNA per reaction well on a StepOnePlus™ Real-Time PCR System (Applied Biosystems, Life Technologies, Carlsbad, CA). Gene expression data was normalized to the 18S rRNA housekeeping gene and calibrated to control samples according to the ΔΔC_T_ methods.

### Statistical analysis

Pairwise comparisons tested for differences from pre-FS supplementation. Paired t-tests (at defined α < 0.05), comparing plasma lignans, oxidative stress biomarkers, plasma cytokines, and buccal epithelium antioxidant mRNA levels were performed for both healthy volunteers and all CF patients evaluating FS-induced changes from baseline within each respective cohort. Upon evaluating individual CF patient levels of plasma lignans, we separated all CF patients (n = 10) into cohorts with low plasma lignans (n = 6) and high plasma lignans (n = 4). This decision was made, post-hoc, as some patients had undetectable levels, while others had levels similar to healthy controls. Pairwise comparisons were then performed on these groups. Paired t-tests or their non-parametric equivalents, Fisher’s exact test and box plots were generated using SAS 9.3 and Stata data analysis and statistical software (release 12, Stata Corp, College Station, TX). No corrections were made for multiple comparisons, as only a few planned comparisons within separate cohorts were made. Additionally this was a small pilot study – hypothesis-generating for future work and such corrections would not change the conclusion of the study.

## Results

15 subjects were enrolled over 3 months and consumed 40 g of finely-ground FS for 4 weeks. Table 1 shows the patient demographics of the CF group. The healthy controls were comparable in age and had no uncontrolled medical problems.

**Table 1:** **Adult Cystic Fibrosis Patient Characteristics**

**Adult Cystic Fibrosis Patient Characteristics**	
Cystic Fibrosis Patients (N)	10
Females (N)	7
Males (N)	3
Age (years) (mean ± SD, range)	31.9 ± 10.8 (23–52)
FEV_1_ (%) (mean ± SD)	76.8 ± 16.8
Body Mass Index (kg/m^2^) (mean ± SD)	22.0 ± 2.8
Pancreatic insufficiency (N)	7
Taking Pancrealipase (N)	8

### Flaxseed safety and tolerability

**Figure 3:**
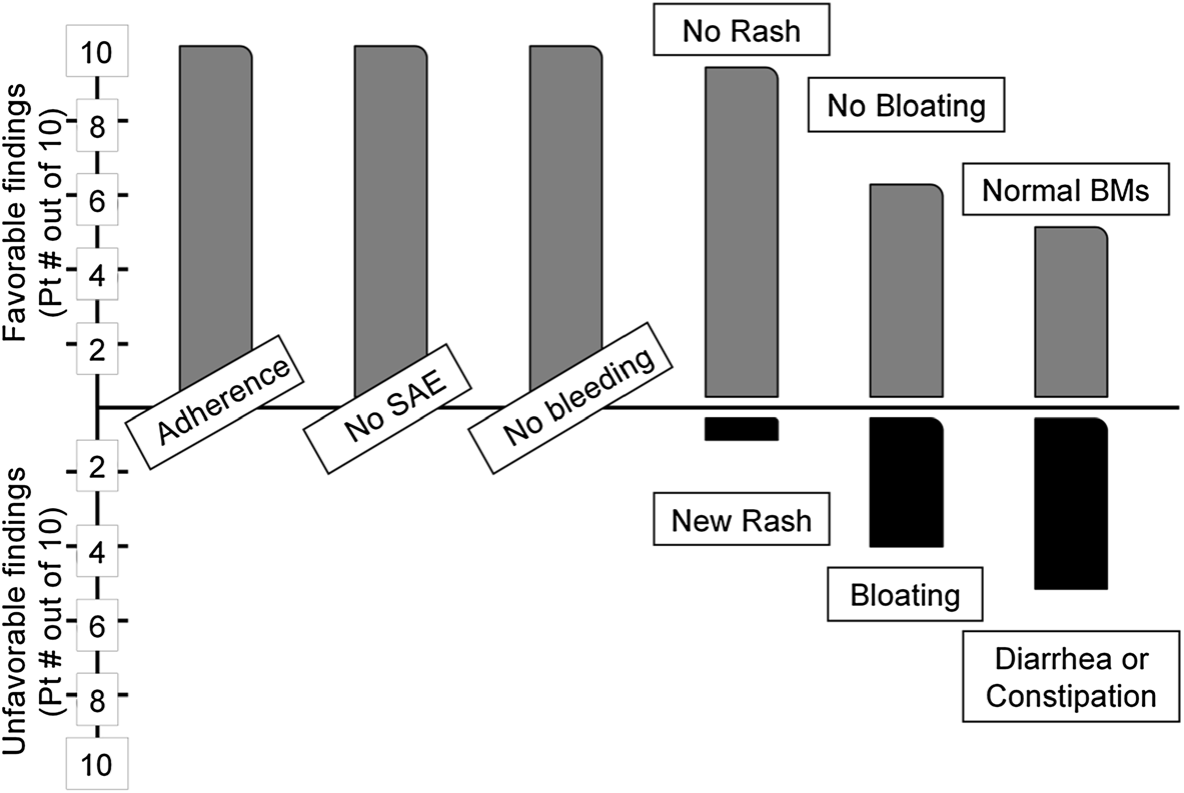
Safety and Tolerability of FS in Adult Cystic Fibrosis Patients. A five question survey was conducted on each clinic visit to assess: 1) adherence to the sample diet; 2) methodology of ingestion, i.e. as smoothie, condiment, etc.; 3) presence or absence of adverse events or other side effects; 4) changes in bowel habits (a known side effect of large volume flaxseed ingestion; and 5) subjective evaluation of mood and perception of wellness, How do you feel today compared to before starting flaxseed…”

Figure 3 shows Safety and Tolerability of FS. All subjects completed the study. No serious adverse effects directly related to FS were observed. No CF exacerbations, hospital admissions, bleeding, anaphylaxis or death occurred while supplementing with FS. A CF patient experienced new onset connective tissue disease during the pilot; however, this flare occurred 1 month after FS discontinuation so was deemed not directly secondary to FS ingestion. The only complaint during supplementation was change in bowel habits. Patients and controls experienced symptoms ranging from mild abdominal discomfort to occasional diarrhea or constipation. Side effects varied, occurred early in supplementation and were self-limited.

### Plasma enterolignan level comparisons

**Figure 4:**
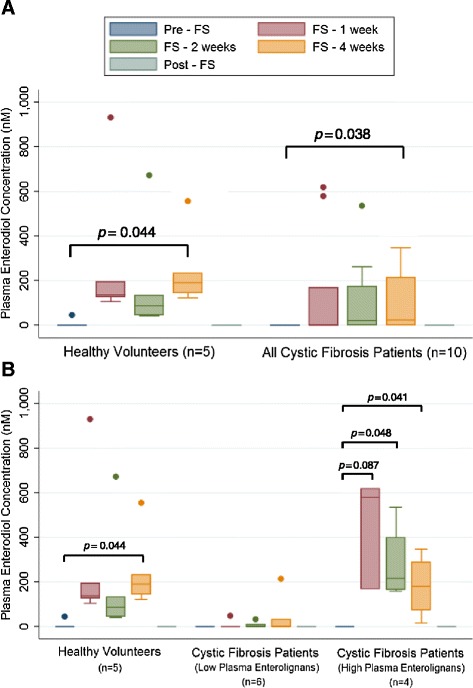
Detection of Enterodiol in Plasma. Levels of plasma enterodiol were quantitated at weeks 0, 1, 2, 4, and 8 of the trial using LC/MS/MS. No enterodiol was detected at week 0, prior to start of trial, and at week 8, which corresponds to 4 weeks after FS was discontinued. Data is presented as plasma concentration (nM). Panel **A** displays comparison between healthy controls (n = 5) and all cystic fibrosis patients (n = 10). Panel **B** displays comparison between healthy controls (n = 5), cystic fibrosis patients with low lignans (n = 6), and cystic fibrosis patients with high lignans (n = 4). * signifies *p* < 0.05 for comparison between any time point in each respective cohort versus baseline (pre-FS).

**Figure 5:**
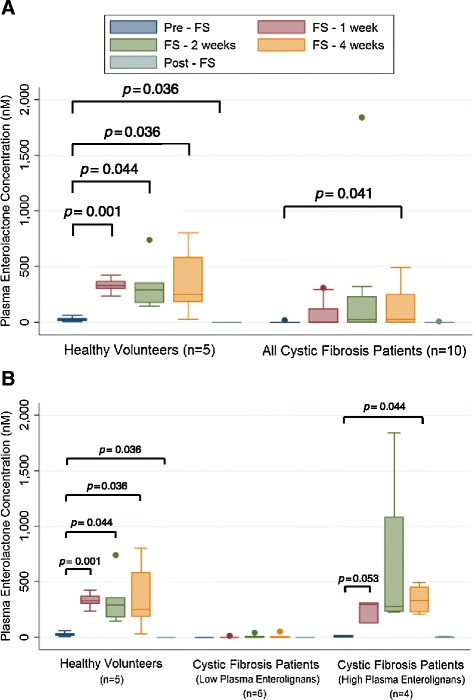
Detection of Enterolactone in Plasma. Levels of plasma enterolactone were quantitated at weeks 0, 1, 2, 4, and 8 of the trial using LC/MS/MS. No enterolactone was detected at week 0, prior to start of trial, and at week 8, which corresponds to 4 weeks after FS was discontinued. Data is presented as plasma concentration (nM). Panel **A** displays comparison between healthy controls (n = 5) and all cystic fibrosis patients (n = 10). Panel **B** displays comparison between healthy controls (n = 5), cystic fibrosis patients with low lignans (n = 6), and cystic fibrosis patients with high lignans (n = 4). * signifies *p* < 0.05 for comparison between any time point in each respective cohort versus baseline (pre-FS). # signifies *p* < 0.05 for comparison between any time point in each respective cohort versus baseline (pre-FS), in which mean is less than baseline.

Figures 4 and 5 display plasma ED and EL levels, respectively. Levels of ED and EL were below the limit of detection prior to study (week 0) and 4 weeks after FS was discontinued (week 8). Figures 4A and 5A compare healthy controls (n = 5) and all CF patients (n = 10). A statistically significant increase from pre-FS baseline in ED and EL levels (*p* = 0.044 and 0.036 respectively) was seen among healthy volunteers following 4 weeks of supplementation. Likewise, the CF population (n = 10) had a statistically significant increase (*p* = 0.038 and 0.041 respectively) in ED and EL levels after 4 weeks. Figures 4B and 5B compare healthy controls (n = 5), CF patients with “low” plasma enterolignan levels (n = 6), and CF patients with “high” plasma enterolignan levels (n = 4). The 6 “low” CF patients did not significantly increase their plasma levels during supplementation. The 4 “high” CF patients statistically significantly increased enterolignan levels (*p* = 0.041 and 0.044 respectively) by 4 weeks.

We uncovered trends when attempting to explain why some patients increased their lignan levels while others did not, shown in Table 2. CF patients with elevated plasma enterolignans were more likely to be older, have lower sweat test and less likely to be diabetic. Additionally, all of the 6 patients with low levels of enterolignans were taking chronic antibiotics (azithromycin or erythromycin), while none of the 4 patients with high levels of enterolignans were on chronic antibiotics.

**Table 2:** **Baseline differences between high and low serum lignan CF patients on flaxseed diet**

	**High lignans (n = 4)**	**Low lignans (n = 6)**	***p*** **-value**
Gender (male)	2 (50%)	1 (17%)	>0.1
Age (years)	38.8 ± 13.8	27.3 ± 5.6	0.099
Sweat chloride (mM)	74.3 ± 23.6	97.6 ± 12.0	0.070
DF508 homozygote (yes)	0 (0%)	3 (50%)	>0.1
Weight (lbs)	161.0 ± 40.1	120.8 ± 18.9	0.062
BMI (Kg/M^2^)	23.2 ± 3.9	21.2 ± 1.8	>0.1
FEV1 (%predicted)	82.8 ± 9.5	72.8 ± 20.2	>0.1
Hemoglobin A1c	5.5 ± 0.2	6.8 ± 1.1	0.051
CF related diabetes (yes)	0 (0%)	4 (66.7%)	0.076
Pancreatic insufficiency (yes)	2 (50%)	6 (100%)	>0.1
Macrolide use (yes)	0 (0%)	6 (100%)	0.005
Mucoid Pseudomonas (yes)	2 (50%)	3 (50%)	>0.1
Non-mucoid Pseudomonas (yes)	2 (50%)	5 (83.3%)	>0.1

### Oxidative stress biomarker modulation

**Figure 6:**
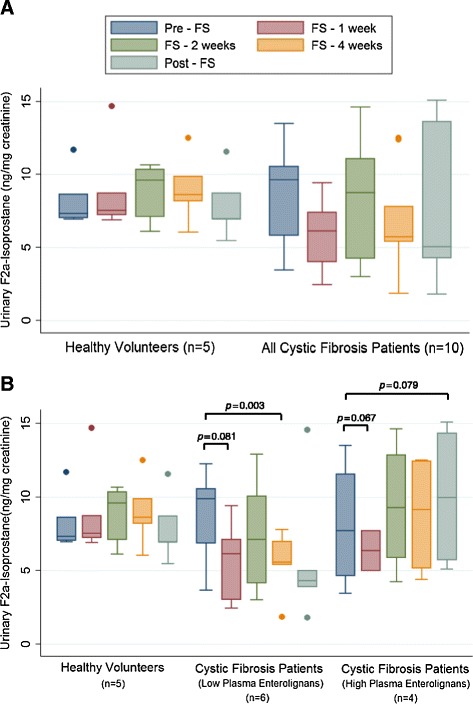
Effect of FS on Urinary F2a-Isoprostane Levels as a Biomarker of Systemic Oxidative Stress. F2a-isoprostane levels, a key biomarker of oxidative stress, were determined from urine samples collected during and after FS supplementation at week 0, 1, 2, 4, and 8. Levels were determined by high-performance liquid chromatography-electrospray tandem mass spectrometry and normalized to urinary creatinine. Panel **A** displays comparison between healthy controls (n = 5) and all cystic fibrosis patients (n = 10). Panel **B** displays comparison between healthy controls (n = 5), cystic fibrosis patients with low lignans (n = 6), and cystic fibrosis patients with high lignans (n = 4). * signifies *p* < 0.05 for comparison between any time point in each respective cohort versus baseline (pre-FS).

**Figure 7:**
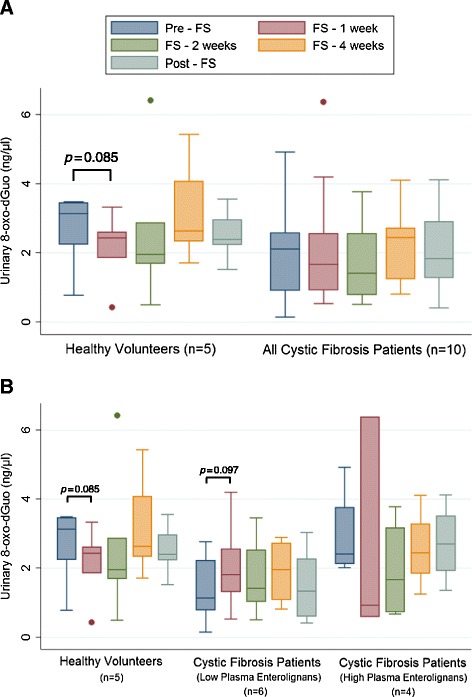
Effect of FS on Levels of Urinary 8-oxo-dGuo as a Biomarker of Systemic Oxidative Stress. 8-oxo-dGuo, one of the major products of DNA oxidation resulting from oxidative stress, was determined from urine samples collected during and after FS supplementation at week 0, 1, 2, 4, and 8. Levels were determined by high-performance liquid chromatography-electrospray tandem mass spectrometry and normalized to urinary creatinine. Panel **A** displays comparison between healthy controls (n = 5) and all cystic fibrosis patients (n = 10). Panel **B** displays comparison between healthy controls (n = 5), cystic fibrosis patients with low lignans (n = 6), and cystic fibrosis patients with high lignans (n = 4).

Figure 6A compares average healthy control IsoP levels to average CF patient IsoP levels. IsoP levels trended from 8.24 ± 1.03 ng to 6.56 ± 0.99 ng after 4 weeks of supplementation (*p* = 0.064). Figure 6B compares average IsoP levels among the controls and the aforementioned cohorts of CF patients with high versus low lignans. CF patients with low lignan levels statistically significantly dropped their IsoP levels from baseline (*p* = 0.003) after 4 weeks of FS administration. A nonsignificant trend was seen for patients with high plasma lignan levels. Levels of 8-oxo-dGuo are shown in Figure 7; no statistically significant differences were seen between healthy controls and the CF patients as a whole (Figure 7A) or in the high and low lignan subgroups (Figure 7B).

### Inflammatory cytokine and immunomodulation

**Figure 8:**
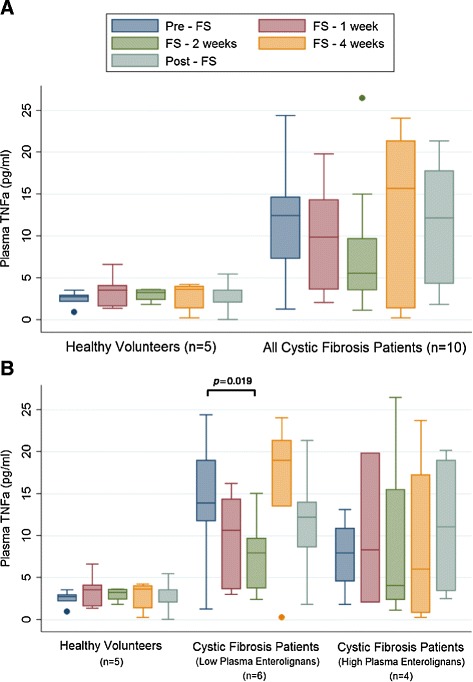
Effect of FS on Levels of Plasma Pro-Inflammatory TNFα. Levels of pro-inflammatory cytokine TNFα were determined from plasma samples collected during and after FS supplementation at week 0, 1, 2, 4, and 8. Panel **A** displays comparison between healthy controls (n = 5) and all cystic fibrosis patients (n = 10). Panel **B** displays comparison between healthy controls (n = 5), cystic fibrosis patients with low lignans (n = 6), and cystic fibrosis patients with high lignans (n = 4). * signifies *p* < 0.05 for comparison between any time point in each respective cohort versus baseline (pre-FS).

**Figure 9:**
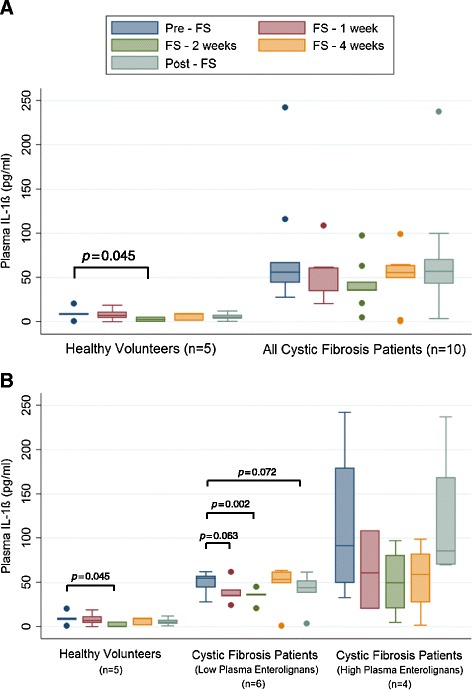
Effect of FS on Levels of Plasma Pro-Inflammatory Interleukin-1β. Levels of pro-inflammatory cytokine IL-1β involved were determined from plasma samples collected during and after FS supplementation at week 0, 1, 2, 4, and 8. Values are reported as pg per ml of plasma. Panel **A** displays comparison between healthy controls (n = 5) and all cystic fibrosis patients (n = 10). Panel **B** displays comparison between healthy controls (n = 5), cystic fibrosis patients with low lignans (n = 6), and cystic fibrosis patients with high lignans (n = 4). * signifies *p* < 0.05 for comparison between any time point in each respective cohort versus baseline (pre-FS).

Figures 8A and 9A compare average plasma TNFα and IL-1ß levels between control and CF volunteers. Overall, the CF patients had higher average plasma TNFα. Figure 8B compares TNFα in controls versus high lignan versus low lignan cohorts. CF patients with low lignan levels statistically significantly dropped (*p =* 0.0019) TNFα levels after 2 weeks of FS, which was not observed among the high lignan CF patients. Similarly, plasma levels of IL-1ß were significantly decreased after 2 weeks of FS administration in CF patients with low lignan levels (Figure 9A).

### Gene expression modulation

**Figure 10:**
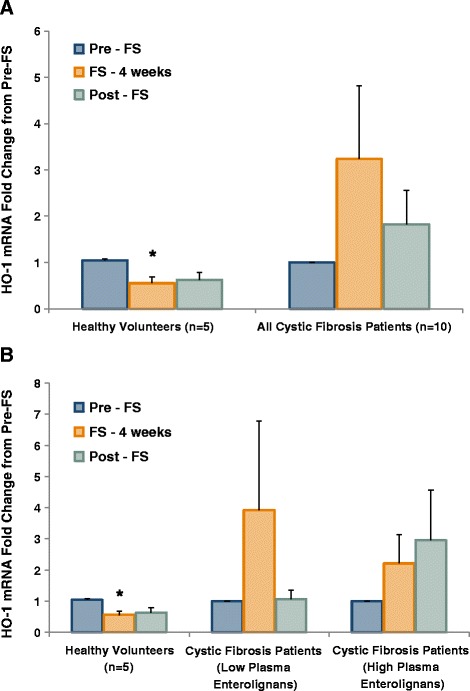
FS Modifies Heme Oxygenase 1 Gene Expression in Buccal Epithelium. Buccal swabs were performed at week 0, 4, and 8 during the study. Buccal epithelial cells were harvested for RNA isolation and subsequent qPCR analysis. Antioxidant gene expression levels were determined using Taqman specific primers and probes to HO-1. Values are reported as fold change from pre-FS. Panel **A** displays comparison between healthy controls (n = 5) and all cystic fibrosis patients (n = 10). Panel **B** displays comparison between healthy controls (n = 5), cystic fibrosis patients with low lignans (n = 6), and cystic fibrosis patients with high lignans (n = 4). * signifies *p* < 0.05 for comparison between any time point in each respective cohort versus baseline (pre-FS).

**Figure 11:**
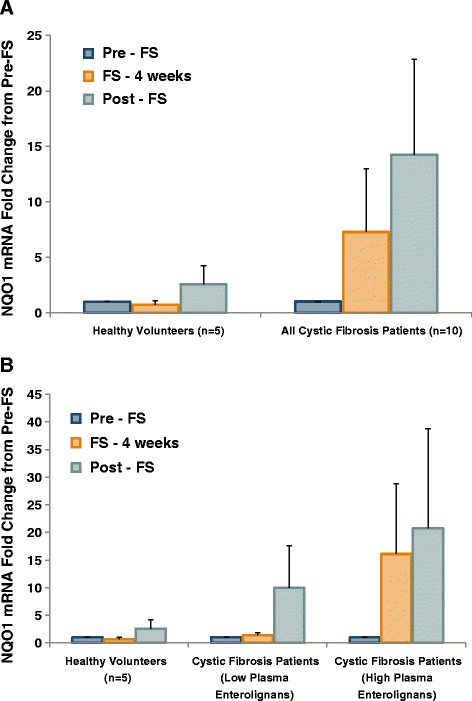
FS Modifies NAD(P)H Quinone Oxidoreductase Gene Expression in Buccal Epithelium. Buccal swabs were performed at weeks 0, 4, and 8 during the study. Buccal epithelial cells were harvested for RNA isolation and subsequent qPCR analysis. Antioxidant gene expression levels were determined using Taqman specific primers and probes to Nqo1. Values are reported as fold change from pre-FS. Panel **A** displays comparison between healthy controls (n = 5) and all cystic fibrosis patients (n = 10). Panel **B** displays comparison between healthy controls (n = 5), cystic fibrosis patients with low lignans (n = 6), and cystic fibrosis patients with high lignans (n = 4).

Figure 10A compares average HO-1 mRNA levels pre-FS, after 4 weeks of FS and post-FS between the controls and CF patients. Notably, levels of HO-1 mRNA were statistically significantly decreased (*p* = 0.026) relative to healthy controls, after 4 weeks on FS, while HO-1 mRNA levels were non-significantly increased 3.24-fold in CF patients after FS administration. Figure 10B compares fold-change in HO-1 levels among healthy control versus CF patients further categorized based on plasma lignan levels. Low lignan CF patients had a 4.57-fold change in HO-1 compared with a 1.90-fold change in high lignan CF patients. No statistical significance was found in these comparisons. Figure 11 shows changes in mRNA levels of NQO1, which however showed no statistically significant changes among controls and CF patients overall (Figure 11A) and stratified by lignan level status (Figure 11B).

## Discussion

This study validated the safety and tolerability of FS in stable CF patients. We demonstrated FS could be safely added to the diet of CF patients without adding unwanted side effects or causing exacerbations. 100% completion of the trial occurred with no SAEs. The primary side effect (well documented) was change in gut motility. CF patients were split into cohorts based on plasma enterolignan levels. The low lignan group demonstrated statistically significant decreases in oxidative stress and inflammatory biomarkers along with upregulation of phase II detoxification/antioxidant enzymes.

Our results differed the ones seen by Milby et al. in lung cancer patients undergoing radiotherapy (XRT), as they consumed FS muffins [28]. They likewise studied FS safety and tolerability while measuring biomarkers of oxidative stress and inflammation. Our population, having the freedom to ingest FS however and whenever they chose could divide FS throughout the day mixing it in shakes, smoothies, etc. thus maximizing tolerability. In contrast, administering dry texture FS muffins seemed to reduce tolerability among oncology patients with xerostomia in Milby *et.al*. Oxidative stress biomarkers also trended lower in this trial.

Intact gut flora facilitates metabolism of FS enriched in secoisolariciresinol diglucoside (SDG) to ED and EL [20]. Malabsorption due to pancreatic insufficiency might have played a role in lower lignan levels. 7 of 10 patients were maintained on azithromycin therapy (initiated long prior to enrollment in this study), likely having reached homeostasis with enteric bacteria. Azithromycin alone would not have eradicated gut flora. SDG is rapidly converted by human intestinal microbiota to SECO and further to the mammalian lignans ED and EL, and is excreted in urine and feces. While no SDG was detected in the plasma of controls and CF patients, FS consumption has been shown to correlate with accumulation of ED and EL levels in plasma [29-31].

Urinary biomarkers of systemic oxidative stress trended lower in CF patients, particularly those who had documented low lignan levels throughout the study. The most dramatic change for nearly every CF patient occurred after that first week of supplementation. The changes among the healthy controls were less obvious. Notably, CF patients were considered “well” or in a steady state in terms of disease, so oxidative stress biomarkers were nearly identical to their healthy control counterparts enrolled in the study. Importantly, the presence of FS did not result in increased oxidative stress on a cellular or DNA level.

The novel data gathered from the human cytokine multiplex samples has been hypothesis generating. This pilot study of 10 highly selected CF patients from a single center showed favorable trends in inflammatory biomarkers particularly in CF patients who demonstrated low lignan levels leading us to ponder how this happened. The inflammatory response of the innate immune system is triggered by a TNF-α surge causing neutrophils, macrophages, and other professional antigen presenting cells to perform their primary functions then present antigen to the adaptive immune system. Chronic inflammation in CF would be dominated by TNF-α, IFN-γ, IL-6, and IL-8 with decreased levels of IL-10, IL-4, etc. Absolute plasma cytokine levels were low (pg/ml), but trends toward decreasing pro-inflammatory cytokine levels were observed among CF patients. Whether this is biologically relevant requires more study of longer duration and with greater numbers.

One of the most interesting findings of our study was the presence of high and low plasma enterolignan levels in CF. The high lignan group had features that were very similar to healthy controls, while low lignan patients had different response in many measures, but improved markers of oxidative stress. One possible explanation could be FS was not absorbed by the low lignan patients; however, the changes in oxidative stress markers suggest that FS had been absorbed causing systemic effect. An alternative explanation could be higher level of oxidative stress in these patients and therefore faster consumption of the lignans. This hypothesis is strengthened by the comparison between the groups. Low lignan patients had higher sweat tests, were pancreatic insufficient, had genotypes associated with severe disease, with worse spirometry. These patients are expected to have more inflammation as a group based on their features. If true, further studies will be needed to assess optimum FS dose to achieve adequate lignan levels causing appropriate antioxidant effect. Our patient number was too small to make any firm conclusions. These results could be erroneous or by chance. Another issue needing further exploration is macrolide use in that group. It is possible this is related to disease severity, which necessitates its use; other possibilities are interference with lignan levels leading to lower levels. Macrolide use was chronic and not new in any patients. These findings will be further explored in larger studies that are in the planning phases.

Along with direct ROS scavenging [14,15], activation of the Nrf2 pathway and downstream effects may explain FS benefits. Buccal swabs (validated surrogates of respiratory epithelia) [32] were collected from CF patients as was done in prior studies of patients with chronic oxidative stress [33-36] to quantify gene expression change from FS. The process of extracting RNA was cumbersome, with a process standardized as much as possible, but could account for error. Regardless, all patients increased expression of specifically NQO1, part of the Nrf2 family of endogenous antioxidants. Increased NQO1 expression continued even after FS was absent from the diet. How and why this occurred is speculative but FS could have activated long-term oxidative stress protection. Additional study is necessary to clarify this interesting finding.

We are aware of our study limitations. It was a single center, non-blinded, non-randomized trial without a control grain for comparison and without correction for multiple comparisons. There were imbalances in the usage of antibiotics among patients with high and low lignan levels, which might explain the difference in levels, but it also might just be a marker of disease severity. Regardless, we were able to show that in this formulation, FS could be incorporated into the daily medical regimen of CF patients and provide a safe, well-tolerated supplement. Lignan levels and how they may impact oxidative stress and gene expression needs further study.

## Conclusions

In the current proof of concept pilot study we were successful in assessing FS tolerability by CF patients and establishing whether certain biomarkers can be measured. The study was not designed to prove that FS works in mitigating disease which will be the focus of larger trials. Indeed, we are in the planning stages of larger trials to further explore our findings and assess whether FS supplementation can lead to improvement in clinically meaningful outcomes in CF patients.

## Abbreviations

8-oxo-dGuo: 8-oxo-7,8-dihydro-2′deoxyguanosine
ARE: Antioxidant response element
CF: Cystic fibrosis
CFTR: Cystic fibrosis transmembrane receptor
ED: Enterodiol
EL: Enterolactone
FS: Flaxseed
GFR: Glomerular filtration rate
HO-1: Heme oxygenase 1
IRI: Ischemia reperfusion injury
IsoP: 8,12-iso-iPF2α-VI-isoprostane
NQO1: NAD(P)H quinone oxidoreductase 1
Nrf2 or NFE2L2: Nuclear factor erythroid 2-related factor 2
RILI: Radiation induced lung injury
SAE: Serious adverse events
SDG: Secoisolariciresinol diglucoside
